# Learning to Take the Heat: Declines in U.S. Heat-Related Mortality

**DOI:** 10.1289/ehp.122-A220

**Published:** 2014-08-01

**Authors:** Lindsey Konkel

**Affiliations:** Lindsey Konkel is a Worcester, MA–based journalist who reports on science, health, and the environment. She is an editor for *Environmental Health News* and *The Daily Climate*.

Americans are learning to take the heat, literally, according to new research in this issue of *EHP*. Overall, the authors of the new study estimate that the risk of heat-related mortality on hotter-than-usual days was 63% lower in 2005 than it was in 1987.^1^ This may indicate the U.S. population, over time, has become more resilient to temperature changes, although some cities showed greater decreases in risk than others.

However, climate change could contribute to additional deaths in the future, according to lead author Jennifer Bobb of the Harvard School of Public Health. “Heat-related mortality is down, but more extreme heat exposures will lead to higher risk,” she says. “I think it is likely that we will continue to adapt, but the rate of adaptation might change moving forward from what we observed in our data from 1987 to 2005.”

**Figure:**
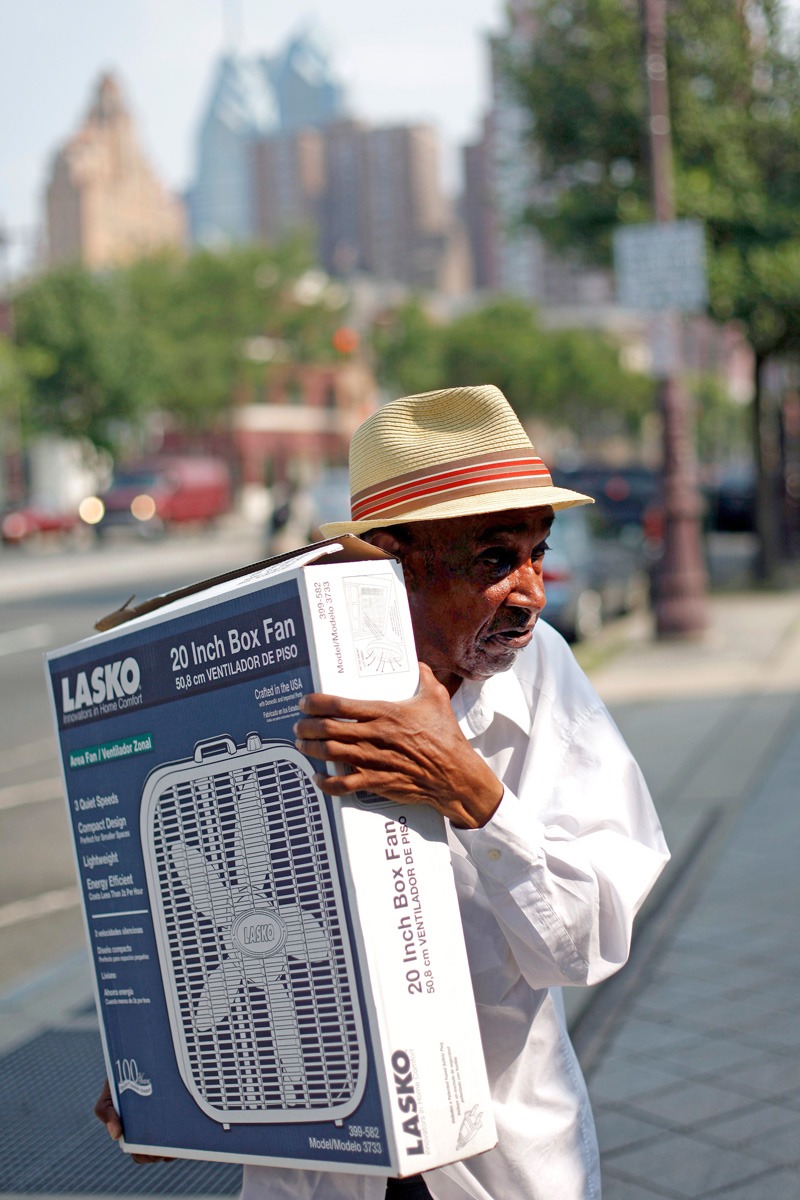
A Philadelphia resident carries a fan distributed by a local senior center. Cities across the United States are implementing public health initiatives to help the elderly and other vulnerable populations cope with hotter-than-usual temperatures in summer months. © AP Photo/Matt Rourke

Bobb and colleagues projected that a 5°F increase in average daily temperatures across the United States would lead to 1,907 additional deaths each summer.^1^ (The National Climate Assessments projects average U.S. temperatures could increase by 3–15°F by 2099, depending on future emissions of greenhouse gases.^2^) The researchers did not account for continued adaptation beyond 2005 in this estimate.

To assess how day-to-day changes in temperature related to changes in daily numbers of deaths, the researchers compared daily summertime weather data for the years 1987 through 2005 with daily deaths reported in 105 U.S. cities (excluding deaths due to external causes, such as accidents and homicides). Their analysis covered about 106 million people.

When they compared mortality on average-temperature days and days with higher-than-average temperatures, the researchers found that the number of excess deaths declined from 51 per 1,000 in 1987 to 19 per 1,000 in 2005. In a sensitivity analysis, they accounted for air pollution levels, which have been linked to increases in same-day mortality risks.^3^ Adults over age 75 and those living in northern cities and cooler climates saw the greatest decrease in estimated heat-related mortality.^1^

Previous studies on heat-related mortality have largely assumed that mortality risk stayed constant over several years.^4^^,^^5^ Fewer studies have investigated how heat-related mortality risk may be changing.^6^ “This study shows that the effect of temperature on deaths isn’t constant over time,” says Patrick Kinney, director of Columbia University’s Climate and Health Program. Kinney was not involved in the study.

A number of factors may account for the decline, according to Bobb. Heat can exacerbate respiratory and cardiovascular diseases,^7^ and cardiovascular deaths make up a large proportion of heat-related deaths.^5^ A decline in heat-related mortality could reflect the overall drop in cardiovascular deaths reported in recent years,^8^ the researchers suggest.

Public health initiatives to increase awareness of the health effects of severe heat also may be working, Bobb says. Several cities have implemented hot-weather warning systems, which include media announcements encouraging people to minimize their own heat exposure and to check on sick and elderly neighbors throughout the day. Cities have also provided “cooling centers” for residents who lack access to air conditioning.^9^

Earlier research suggested that an increase in the use of central air conditioning over the past few decades may be a major contributor to declines in heat-related deaths.^6^^,^^10^ But Bobb and colleagues found that air conditioning may not play as big a role as previously thought.

Although the prevalence of central air conditioning in homes increased by an average of about 1% each year over the study period (from a low of 48% in 1987), the researchers found no evidence of larger declines in heat-related mortality for cities with larger increases in air-conditioning prevalence.^1^ However, their analysis was limited by the unavailability of data on air conditioning usage among different groups.

“What we’d really like to see is how much air conditioning is used by people who are the most vulnerable to the health effects of heat, and that sort of information isn’t readily available,” Kinney says. “My intuition is still that air conditioning may be a key factor.”

Although this report focused solely on heat-related mortality during the months of June, July, and August, the health effects of heat are sometimes most striking early in the season, before people have acclimated to the shift in the seasons, says Kinney. With climate change projected to increase year-round temperatures across months,^2^ he says that including additional months may provide a more comprehensive picture of heat-related mortality risk throughout the year.
